# Proteolytic control of mitochondrial protein translocases

**DOI:** 10.1002/pro.70553

**Published:** 2026-04-19

**Authors:** Lara Kroczek, Thomas Langer

**Affiliations:** ^1^ Department of Mitochondrial Proteostasis Max Planck Institute for Biology of Ageing Cologne Germany; ^2^ Cologne Excellence Cluster on Cellular Stress Responses in Aging‐Associated Diseases (CECAD) University of Cologne Cologne Germany

**Keywords:** mitochondrial proteases, mitochondrial protein import, mitochondrial remodeling, protein quality control

## Abstract

Mitochondria are essential organelles that drive numerous cellular processes, including energy metabolism, ion homeostasis, and programmed cell death. This functional versatility relies on a highly dynamic proteome whose composition is continuously remodeled to meet changing cellular and environmental demands. Central to this remodeling are mitochondrial proteases (termed mitoproteases), which maintain protein quality and regulate mitochondrial function through selective processing and degradation events. Their activity ensures rapid degradation of regulatory proteins and dynamically adjusts components of multiprotein complexes. Among their most critical targets are elements of the mitochondrial protein import machinery. By modulating translocase stability and by processing preproteins during translocation, mitoproteases enable precise control over the organelle's proteome, aligning mitochondrial function with the cell's metabolic state. This review discusses how mitoproteases maintain translocase integrity and dynamically regulate mitochondrial protein import and the mitochondrial proteome.

## 1 INTRODUCTION

Mitochondria emerged more than 1.5 billion years ago through the integration of an ancestral α‐proteobacterium into a proto‐eukaryotic host cell (Martin et al., 2015). This endosymbiotic event marked a decisive step in evolution, establishing eukaryotes as key players in the development of complex life (Martin et al., 2015; Roger et al., 2017; Sagan, 1967). Mitochondria support diverse anabolic and catabolic processes by supplying energy, biosynthetic precursors and key signaling metabolites essential for cellular homeostasis (Monzel et al., 2023; Spinelli & Haigis, 2018). Mitochondrial function is shaped by environmental and metabolic cues that continuously remodel organellar pathways to meet cellular demands. This dynamic adaptability, termed mitochondrial plasticity, reflects the ability of mitochondria to reorganize their proteome in response to changing conditions. It relies on coordinated mitochondrial and cellular protein quality control systems that regulate not only mitochondrial biogenesis but also broader cellular stress responses (Palmer et al., 2021; Song et al., 2021). Central to this regulation are mitoproteases, which fine‐tune mitochondrial pathways through targeted protein degradation or specific protein processing events (Deshwal et al., 2020). Their rapid and selective activity enables remodeling of mitochondrial dynamics, initiation of stress responses, and adjustment of the mitochondrial proteome to maintain cellular homeostasis. The activity of many mitoproteases decline with age and dysregulation of mitochondrial proteases has been linked to neurodegeneration, cardiovascular diseases and cancer, similar to mitochondrial diseases associated with OXPHOS deficiencies (Baker et al., 2025; Deshwal et al., 2020; Ghifari et al., 2025; Gusic & Prokisch, 2021; Lopez‐Otin et al., 2023; Suomalainen & Nunnari, 2024; Wen et al., 2025).

Over evolutionary time, the mitochondrial genome has become highly reduced, making mitochondria dependent on importing most proteins after their synthesis at cytosolic ribosomes. Protein import occurs via conserved, multisubunit protein translocases in both mitochondrial membranes along five principal pathways, which are described in detail in excellent recent reviews (Busch et al., 2023; Endo & Wiedemann, 2025; Jain et al., 2025). The translocase of the outer membrane (TOM) complex serves as the main entry gate for the majority of mitochondrial proteins, before being directed to one of several specialized routes: The translocase of the inner membrane 23 (TIM23) complex for matrix and inner membrane (IM) proteins, the translocase of the inner membrane 22 (TIM22) complex for multispanning metabolite carrier proteins of the IM, the MIA pathway for disulfide‐bonded intermembrane space (IMS) proteins, the mitochondrial sorting and assembly machinery (SAM) complex mediating the insertion of β‐barrel proteins into the outer membrane (OM), or the mitochondrial import machinery (MIM) for α‐helical OM proteins.

Given the essential role of mitochondria for cell homeostasis, it is not surprising that deficiencies in mitochondrial protein import severely affect cell function and survival. Mutations affecting the mitochondrial import machinery are associated with diseases (Jain et al., 2024; Nicolas et al., 2019; Palmer et al., 2021) and protein import stress caused by accumulating mitochondrial preproteins (Calais & Bertolin, 2026; Lionaki et al., 2023; Pfanner et al., 2025; Wang & Chen, 2015; Wrobel et al., 2015). This can induce cellular stress responses, including the integrated stress response (Fessler et al., 2020; Fessler et al., 2022; Guo et al., 2020; Sekine et al., 2023), the mitochondrial unfolded protein response (UPR^mt^) (Sutandy et al., 2023), and mitophagy (Michaelis et al., 2022).

Recent evidence revealed that mitoproteases regulate protein import into mitochondria safeguarding the mitochondrial proteome and adjusting it to physiological demands. Substrates of mitoproteases include mitochondrial precursor proteins during membrane translocation and components of mitochondrial protein translocases, allowing for a dynamic adjustment of the import process. Here, we will review the different strategies through which mitoproteases orchestrate mitochondrial protein import and shape the mitochondrial proteome.

## 2 REGULATION OF PROTEIN LOCALIZATION BY PROTEOLYTIC PROCESSING

Mitochondria harbor an independent proteolytic system that allows the complete degradation of proteins to amino acids (Baker et al., 2025; Deshwal et al., 2020; Szczepanowska & Trifunovic, 2022). Processing peptidases that cleave off mitochondrial targeting sequences (MTSs) from preproteins upon import into mitochondria were among the first proteases identified within mitochondria (Garrido et al., 2022; Gomez‐Fabra Gala & Vogtle, 2021; Kunova et al., 2022). Mitochondria harbor several processing peptidases localized in different mitochondrial compartments, including the general mitochondrial processing peptidase (MPP) and the mitochondrial intermediate peptidase (MIPEP or MIP) in the matrix space and the inner membrane peptidase (IMP) (Kunova et al., 2022). Dysfunction of these processing proteases disrupts proteostatic balance and causes severe mitochondrial disease (Choquet et al., 2016; Eldomery et al., 2016; Jain et al., 2025; Jobling et al., 2015; Joshi et al., 2016; Nicolas et al., 2019; Palmer et al., 2021; Vögtle et al., 2018).

Maturation of mitochondrial preproteins is usually required for their functionality but can also affect their cellular localization. Yeast fumarase, which is targeted to the mitochondrial matrix by a MTS, cleaved off by MPP, was the first described example for a folding‐driven retranslocation and partitioning between two cellular compartments (Sass et al., 2001; Sass et al., 2003). After initial targeting to mitochondria and partial passage through the translocase, the fumarase accumulates as an import intermediate that can either be completely imported into the matrix or return to the cytosol. The direction of movement is dictated by the protein folding state: folding of the N‐terminal region promotes complete import into the matrix, whereas folding of the C‐terminal region on the cytosolic side blocks further translocation and drives retrograde movement back into the cytosol (Peleg et al., 1990; Stein et al., 1994). This folding‐controlled bidirectional passage through the pore provides a conceptual framework for proteins that distribute between mitochondria and cytoplasm, depending on import‐coupled conformational changes, on alterations in the mitochondrial membrane potential or on proteolytic processing (Pines et al., 2024).

**FIGURE 1:**
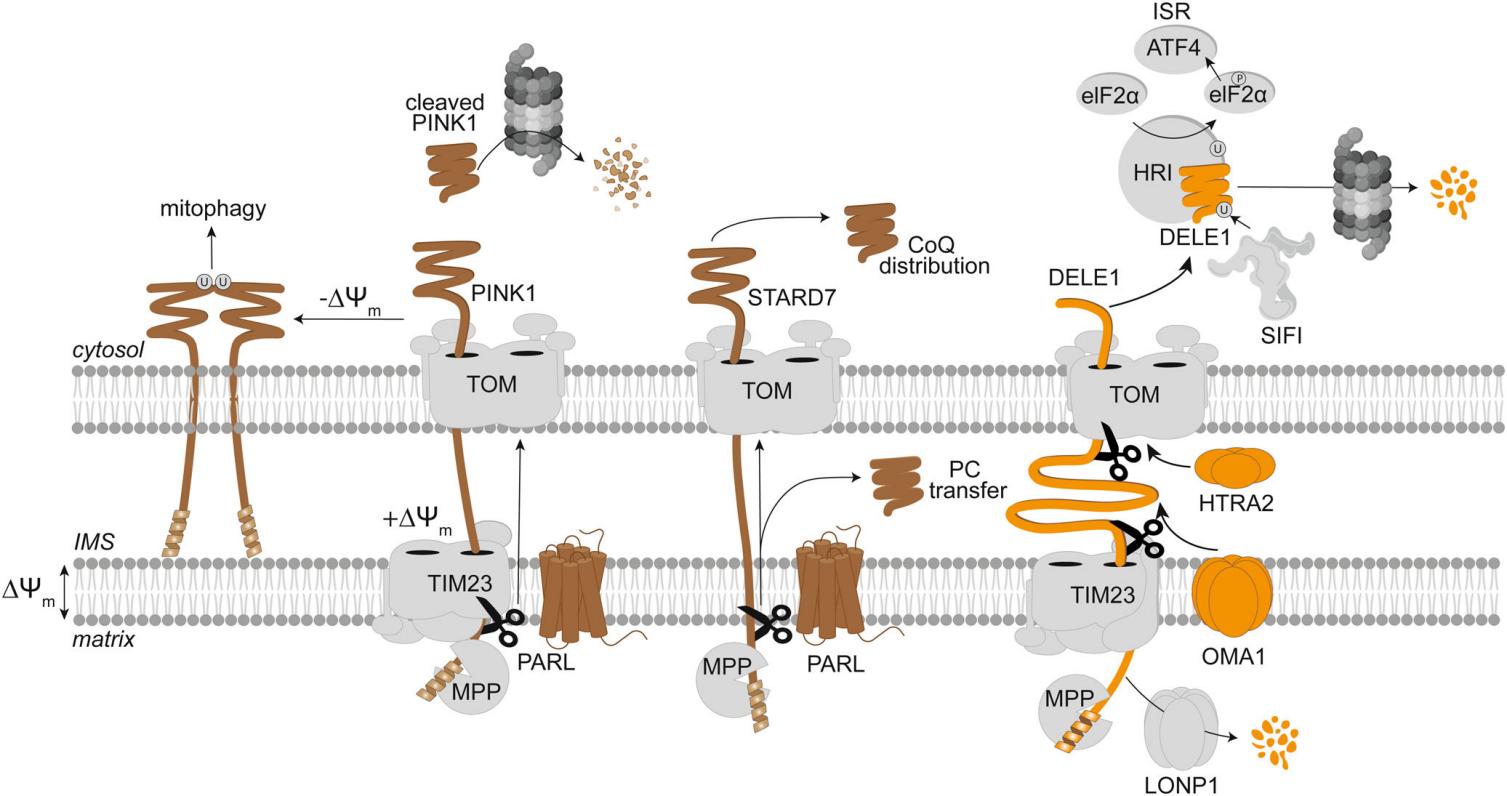
Proteolytic processing of mitochondrial proteins during translocation determines their fate and function in humans. The mitochondrial processing peptidase (MPP) cleaves off the mitochondrial targeting sequences of PINK1 (PDB: 9EIH), STARD7 and DELE1 (PDB: 8D9X) in the matrix. PINK1 is additionally cleaved by PARL in the IM, leading to retro‐translocation and proteasomal degradation in the cytosol. When the mitochondrial membrane potential is impaired, PINK1 accumulates on the OM, triggering mitophagy. The lipid transfer protein STARD7 undergoes processing by PARL within the IM, generating two distinct STARD7 pools. IMS‐localized STARD7 serves as a lipid transfer protein for phosphatidylcholine (PC), supporting mitochondrial structure and function, including coenzyme Q (CoQ) biosynthesis, while cytosolic STARD7 supports CoQ transport to the plasma membrane and ferroptotic resistance. DELE1 acts as a mitochondrial stress sensor that is continuously degraded by the matrix protease LONP1 under basal conditions. Upon mitochondrial stress, OMA1 or HTRA2 (PDB: 1LCY) cleave DELE1 during import, generating different short forms that localize to the cytosol and activate the ISR^mt^.

The rhomboid protease PARL, an intramembrane‐cleaving serine peptidase in the IM (Adrain & Cavadas, 2020; Gourkanti et al., 2025; Spinazzi & DE Strooper, 2016), exemplifies how a protease can affect the cellular localization of their substrates by proteolytic cleavage. Several known substrates of PARL were found to be dually localized depending on PARL processing (Saita et al., 2017). PARL mediates the maturation of Smac/DIABLO in the IMS, which is a prerequisite for its release into the cytosol upon initiation of apoptosis (Saita et al., 2017). Another substrate of PARL, the lipid transfer protein STARD7, is cleaved by PARL during its translocation across the IM, followed by the distribution of mature STARD7 to the IMS or, after release from the import pore, to the cytosol (Figure 1). STARD7 serves as a lipid transfer protein for phosphatidylcholine in the IMS maintaining cristae morphogenesis and respiration. Moreover, mitochondrial STARD7 preserves coenzyme Q synthesis, whereas cytosolic STARD7 ensures coenzyme Q transport from mitochondria to the plasma membrane, thus connecting import‐coupled processing to ferroptosis protection (Deshwal et al., 2023; Saita et al., 2018). This exemplifies how PARL‐mediated processing of a single protein can establish functional distinct pools in different compartments of the cells.

PARL has also been linked to the regulation of mitophagy mediated by the PTEN‐induced serine kinase PINK1 and the E3 ubiquitin ligase Parkin (Narendra & Youle, 2024) (Figure 1). PARL‐mediated PINK1 cleavage during import causes the release of cleaved PINK1 from the import pore and its degradation by the cytosolic ubiquitin‐proteasome system (UPS) (Jin et al., 2010). However, mitochondrial depolarization prevents insertion of PINK1 into the IM and PARL cleavage, resulting in the accumulation of PINK1 in the OM. Phosphorylation of ubiquitin promotes Parkin recruitment to the mitochondrial surface, broad ubiquitylation of mitochondrial proteins and initiation of mitophagy. Notably, in depolarized mitochondria, PARL preferentially cleaves the serine–threonine phosphatase PGAM5, which promotes mitophagy by PINK1 stabilization and by dephosphorylating the mitophagy receptor FUNDC1 (Sekine et al., 2012). Thus, PARL cleavage coordinates different mitophagy pathways in response to mitochondrial damage.

Proteolytic processing of mitochondrially targeted proteins during import can also induce stress signaling from dysfunctional mitochondria. The signaling protein DELE1 is targeted to the mitochondrial matrix where it is degraded by the LONP1 protease (Sekine et al., 2023). However, stress conditions, such as membrane potential loss, OXPHOS defects, oxidative, heat or protein stress, activate the peptidase OMA1 in the IM, which acts as both stress sensor and effector (Baker et al., 2014; Bohovych et al., 2014; Bohovych et al., 2019; Kaser et al., 2003; Miallot et al., 2023; Murata et al., 2020; Ohba et al., 2020; Rainbolt et al., 2016; Zhang et al., 2014). Activated OMA1 cleaves DELE1 during its import into mitochondria, allowing the release of cleaved DELE1 into the cytosol (Fessler et al., 2022) (Figure 1). DELE1 binding to the heme‐regulated inhibitor kinase (HRI, EIF2AK1) induces eIF2α phosphorylation and the mitochondrial integrated stress response (ISR^mt^) (Fessler et al., 2020; Guo et al., 2020). Degradation of DELE1 by the SIFI E3 ubiquitin ligase complex and the 26S proteasome system (Haakonsen et al., 2024; Martinez Castillo & Evans, 2024; Yang et al., 2025) and of OMA1, either autocatalytically or by the IM protease YME1L, terminates stress signaling (Baker et al., 2014; MacVicar et al., 2019; Rainbolt et al., 2016).

Studies in mice support the importance of OMA1‐mediated DELE1 cleavage for ISR^mt^ induction in vivo (Ahola et al., 2022; Franchino et al., 2024; Huynh et al., 2023; Lin et al., 2024). However, *Dele1* ablation affects the ISR^mt^ more strongly than the loss of OMA1, indicating that additional proteases are able to cleave DELE1. Indeed, the IMS‐localized serine peptidase HTRA2 was found to cleave DELE1 within its carboxy‐terminal domain in HEK293 cells upon non‐depolarizing import stress, generating a different short form of DELE1 that is still capable to activate the ISR^mt^ (Bi et al., 2024). Moreover, stabilization of non‐cleaved DELE1 at the mitochondrial surface in iron deficiency allows ISR^mt^ induction without proteolytic cleavage by OMA1 (Sekine et al., 2023). Thus, while OMA1 senses mitochondrial deficiencies to induce stress signaling, the critical determinant of ISR activation is the localization and import state of DELE1, rather than the engagement of a single dedicated protease (Bi et al., 2024). Several peptidases, such as PARL, OMA1, and HTRA2, can function as import‐coupled signaling hubs that operate at the interface of import and signaling, generating stress‐specific cytosolic messengers from a mitochondrial precursor.

## 3 THE STABILITY OF PROTEIN TRANSLOCASES

Impaired proteolytic maturation of mitochondrial preproteins upon import into mitochondria can lead to protein aggregation and elicits an unfolded protein response‐like (UPR^mt^‐like) stress response in yeast (Poveda‐Huertes et al., 2020). This is accompanied by an acute stimulation of protein biogenesis, which however ceases if stress persists (Poveda‐Huertes et al., 2021). Therefore, mitoproteases serving as processing peptidases can determine protein localization and affect more broadly the mitochondrial protein import via stress signaling. Increasing evidence suggests that mitoproteases also modulate the mitochondrial proteome by directly regulating mitochondrial protein import (Table 1).

**TABLE 1:** Proteolytic regulation of the mammalian mitochondrial protein import machineries.

Protease	Subcompartment	Substrates	Physiological/cellular context
OMA1	Inner membrane (catalytic site IMS)	DNAJC15, AIFM1	Import limitation of OXPHOS‐related proteins under mitochondrial stress Clearance of clogged import pores
YME1L, i‐AAA protease	Inner membrane (catalytic site IMS)	TIMM22, TIMM17A, TIMM23, ROMO1, TIMM13, TIMM8B	Adjustment of protein import under hypoxia/starvation Clearance of clogged import pores
AFG3L2 (*SPG7*), m‐AAA protease	Inner membrane (catalytic site matrix)	DNAJC15, PAM16, OXA1L	Remodeling of the PAM complex Import limitation of OXPHOS‐related proteins under mitochondrial stress

**FIGURE 2:**
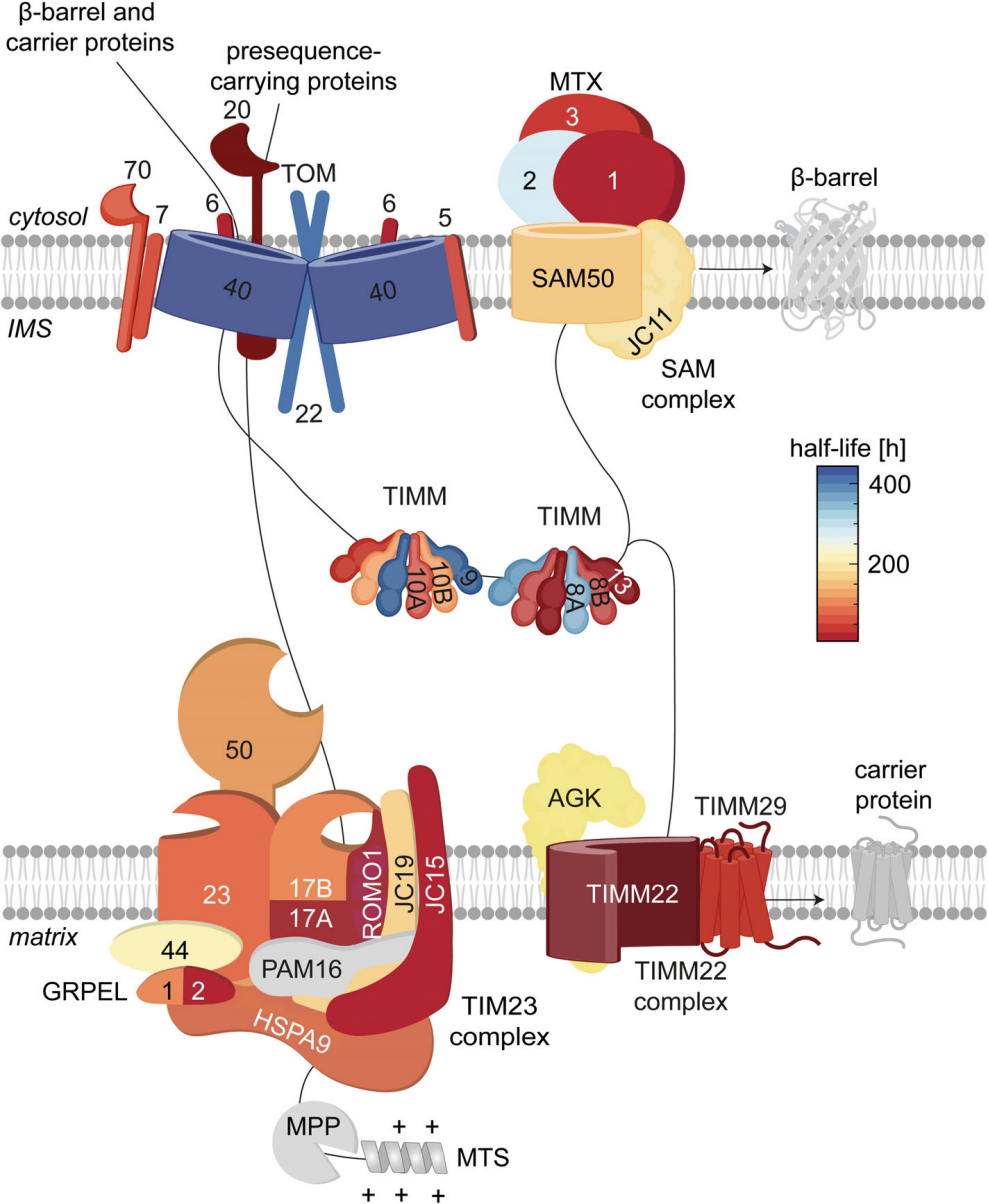
The stability of components of different mitochondrial protein import pathways. Protein half‐lives in human cell lines are shown (Morgenstern et al., 2021). Precursor proteins enter mitochondria via the translocase of the outer membrane (TOM) complex, which contains a stable core and regulatory receptor subunits with shorter half‐lives. β‐barrel proteins are transferred from TOM to the sorting and assembly machinery (SAM) complex, where metaxin (MTX) proteins facilitate their insertion into the OM. TIM23 complexes mediate the import of presequence‐containing proteins into the matrix or IM with MPP cleavage of the mitochondrial targeting sequence (MTS), whereas TIM22 complexes insert metabolite carriers into the IM. The TIM23 machinery in humans includes multiple paralogous subunits with distinct apparent half‐lives, such as the channel‐forming subunits TIMM17A and TIMM17B, the J‐domain‐containing cochaperones DNAJC15 and DNAJC19, and the nucleotide exchange factors GRPEL1 and GRPEL2.

Quantitative proteomics has revealed that subunits within the same protein translocase complex have markedly different apparent half‐lives (Morgenstern et al., 2021) (Figure 2). Long‐lived structural cores coexist with short‐lived regulatory components, suggesting that proteolysis enables the dynamic remodeling of translocase complexes allowing them to adjust import rates and specificities in response to physiological demands. However, we are only at the beginning to define these regulatory circuits and the proteolytic pathways involved in the degradation of protein translocase subunits.

The TOM complex forms the universal entry gate for nuclear‐encoded precursors in the OM. Its pore‐forming core, TOMM40, is remarkably stable, providing a constant structural scaffold. In contrast, the receptor subunits TOMM20 and TOMM70 display shorter apparent half‐lives, supporting adjustable import capacity in response to metabolic demand (Araiso et al., 2019; Araiso & Endo, 2022; Morgenstern et al., 2021; Shiota et al., 2015; Tucker & Park, 2019; Wang et al., 2020). Similarly, in the SAM complex mediating the insertion of β‐barrel proteins into the OM, the core subunit SAM50 exhibits long‐term stability anchoring the complex, whereas its accessory partners MTX1 and MTX3 have shorter half‐lives and likely tune β‐barrel folding and insertion efficiency (Ganesan et al., 2024).

Although additional studies are needed, current evidence indicates that subunits of OM translocase complexes are degraded via the UPS, similar to other OM proteins (Song et al., 2021; Yang et al., 2024). This mechanism involves ubiquitylation by cytosolic E3 ubiquitin ligases and extraction of individual proteins from the OM and therefore explains different turnover rates of subunits of translocase complexes. In *Arabidopsis*, OM translocase components are ubiquitinated by E3 ligases and subsequently extracted by the AAA‐ATPase CDC48 (Li & Jarvis, 2024; Ling et al., 2019; Yang et al., 2024), the plant orthologue of human VCP/p97 (Xu et al., 2011). Similarly, in yeast, components of the SAM complex undergo UPS‐mediated degradation, a process regulated by the ubiquitin ligase Ubr1 and coordinated activity of Hsp70 and Hsp40 chaperones (Metzger et al., 2020).

The selective ubiquitylation of OM proteins by cytosolic E3 ubiquitin ligases and extraction of individual proteins from the OM explains different turnover rates of subunits of translocase complexes. Other mechanisms allow to selectively adjust the level of assembled translocase complexes in the OM, independent of the degradation of the entire organelle by mitophagy. Assembled TOM complexes can be transported in mitochondria‐derived vesicles to lysosomes (König et al., 2021). SAMM50, the core subunit of SAM complexes contains a LIR motif and can interact with ATG8 and p62 proteins to induce a piecemeal mitophagy, leading to the degradation of SAM complexes and of the mitochondrial contact site and cristae organizing system (MICOS complexes) without the involvement of mitochondria‐derived vesicles (MDVs) (Abudu et al., 2021).

After passage through the TOM complex small TIM chaperones (TIMM8‐TIMM13; TIMM9‐TIMM10) support the biogenesis of β‐barrel proteins in the OM and shuttle metabolite carrier proteins across the IMS before being inserted into the IM via TIM22 complexes. In humans, the six small TIM proteins TIMM8A, TIMM8B, TIMM9, TIMM10A, TIMM10B, and TIMM13 (Anderson et al., 2023) have different half‐lives, which suggests functional specialization. TIMM8A and TIMM9 are relatively stable, while TIMM8B, TIMM10A/B and TIMM13 have shorter half‐lives (Morgenstern et al., 2021). Small TIM proteins are primarily degraded by the i‐AAA protease YME1L (Baker et al., 2012), an ATP‐dependent proteolytic complex in the IM that exposes its catalytic domain to the IMS (Ohba et al., 2020). Notably, the short‐lived proteins TIMM13 and TIMM8B are targeted by YME1L under stress conditions such as hypoxia and starvation (MacVicar et al., 2019). Yeast Yme1 selectively recognizes destabilized or non‐assembled TIM proteins. Accordingly, Tim9 and Tim10 assembly into hexameric complexes prevents the degradation of Tim10 by Yme1 (Spiller et al., 2015). Interestingly, Yme1 preferentially binds Tim10 over the other small TIM proteins and also interacts with assembled Tim9‐10 complexes (Quispe‐Carbajal et al., 2026). However, degradation occurs only upon disruption of the internal disulfide bond in Tim10, which induces a conformational change and exposes an amino‐terminal region recognized by Yme1.

Different multimeric protein translocases, the TIM22 and the TIM23 complex, mediate the insertion into or translocation of proteins across the IM. The TIM22 complex, composed of TIMM22, TIMM29 and the acylglycerol kinase AGK, facilitates the insertion of metabolite carrier proteins in the IM. AGK ensures the stability of the TIM22 complex, consistent with its longer half‐life, whereas the core components of the TIM22 machinery, TIMM22 and TIMM29, are degraded more rapidly, which may allow to adjust the import capacity to metabolic demands. Accelerated degradation of TIMM22 by the i‐AAA protease YME1L was observed in hypoxia or in starved cells (MacVicar et al., 2019). In yeast, genetic evidence links the functional integrity of the TIM22 complex to the i‐AAA protease Yme1 (Kumar et al., 2023). Loss of Yme1 destabilizes the TIM22 complex, and concurrently targets TOM complex components such as Tom22 for degradation (Kan et al., 2022; Wu et al., 2018).

**FIGURE 3:**
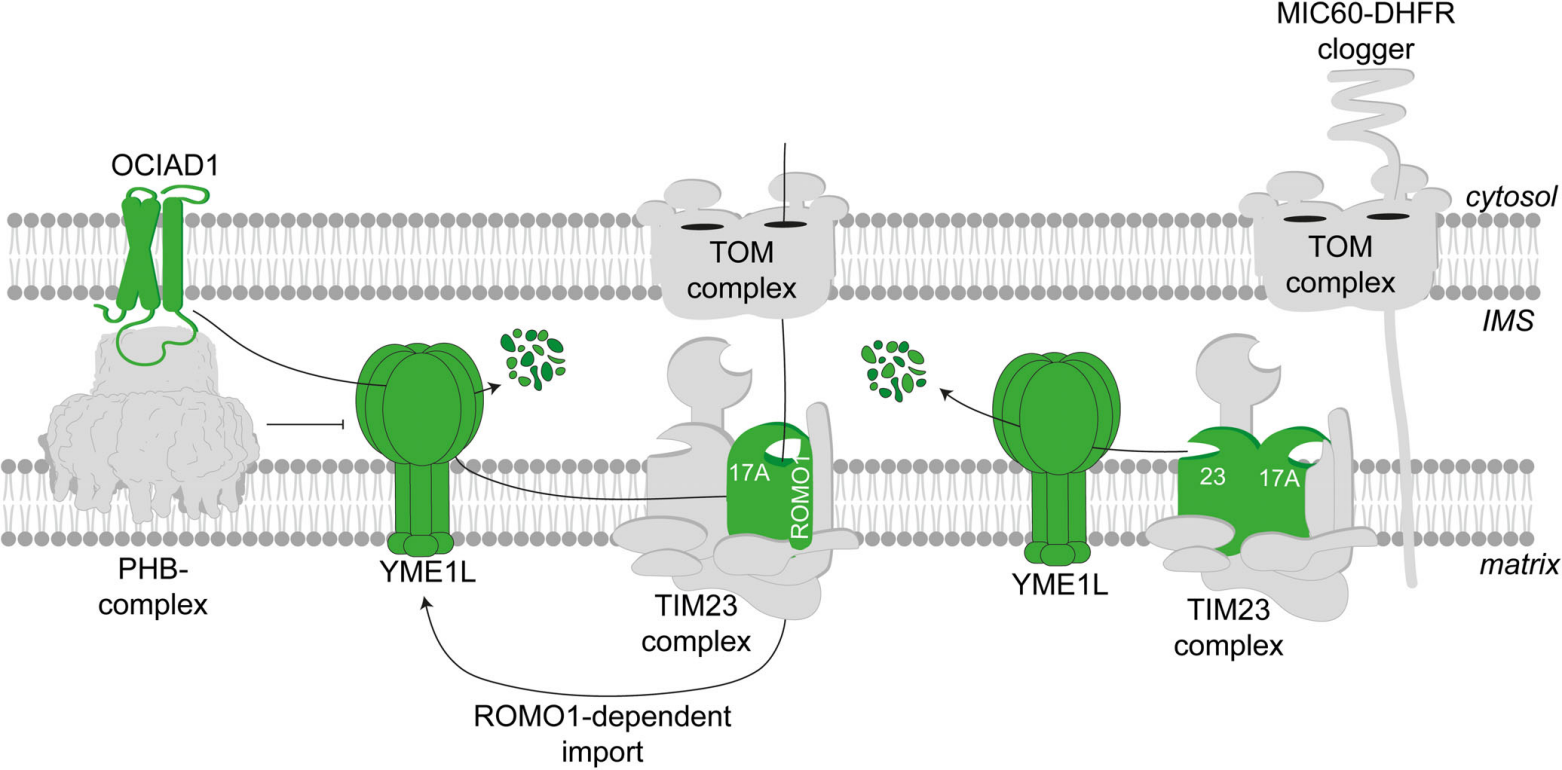
YME1L‐mediated remodeling of the TIM23 complex. The IM protease YME1L recognizes and degrades the short‐lived core subunit TIMM17A, thereby modulating activity of the translocation machinery. In addition, YME1L targets ROMO1, which regulates IM insertion via the TIM23 complex and is also required for YME1L insertion in the IM. YME1L activity is regulated by OCIAD1, which associates with the prohibitin (PHB) membrane scaffold complex (PDB: 8RRH) and suppresses YME1L activity. Upon clogging of the TOM complex, YME1L selectively degrades TIMM17A and TIMM23 subunits, preventing precursor accumulation at the TIM23 complex.

These findings highlight the central role of YME1L‐mediated proteolysis for the regulation of mitochondrial import and proteostasis under stress. Indeed, YME1L‐mediated proteolysis affects also the TIM23 complex, whose subunits show remarkably different stabilities, allowing rapid post‐translational alterations in TIM23 complexes and mitochondrial protein import (Figure 3).

## 4 PROTEOLYTIC REMODELING OF TIM23 COMPLEXES UNDER STRESS

The TIM23 complex allows the translocation of mitochondrial preproteins across the IM into the matrix or their lateral insertion into the IM. TIMM23 interacts with TIMM17 proteins, which forms a laterally open pore sealed by ROMO1 (or Mgr2 in yeast) (Fielden et al., 2023; Sim et al., 2023). Two paralogs of TIMM17 exist in mammalian mitochondria: TIMM17B constitutes a stable channel‐forming subunit, while TIMM17A is short‐lived and rapidly degraded by YME1L (Morgenstern et al., 2021; Rainbolt et al., 2013). The carboxy‐terminal domain of TIMM17A harbors a degron that is recognized specifically by YME1L and is absent in TIMM17B (Hsu et al., 2025). In hypoxia or in starved cells, mTORC1 inhibition induces a LIPIN1‐dependent phospholipid signaling cascade that ultimately leads to decreased phosphatidylethanolamine (PE) levels in the IM and activates YME1L‐mediated proteolysis (MacVicar et al., 2019). YME1L selectively degrades metabolic enzymes, lipid transfer proteins and components of the protein import machinery, including TIMM17A, TIMM23, ROMO1, TIMM22, and small TIM proteins, thereby restricting mitochondrial biogenesis and shifting organellar metabolism to sustain cell viability under stress (MacVicar et al., 2019) (Figure 3). Similarly, translational attenuation upon phosphorylation of eIF2α under cellular stress leads to the degradation of TIMM17A by YME1L. It is presently unclear whether this is the consequence of ongoing proteolysis combined with an impaired TIMM17A synthesis or whether TMM17A degradation is increased under these conditions. Regardless, TIMM17A proteolysis limits the import of nuclear‐encoded proteins and elicits a mitochondrial UPR in *Caenorhabditis elegans*, alleviating the protein load on organellar proteostasis (Rainbolt et al., 2013). The attenuation of import capacity is reminiscent of observations in yeast, where Yme1 orchestrates degradation of Tim17, Tim23, and Tim44 to fine‐tune the TIM23 complex (Kan et al., 2022). Although these findings identify YME1L as a central effector and integrator of mitochondrial stress signaling, it remains to be determined how the turnover of selective TIM23 complex subunits affects the mitochondrial proteome, either by merely reducing the import capacity or by altering the composition and specificity of TIM23 complexes.

The turnover of TIMM17A by YME1L is tightly controlled. The ovarian cancer immunoreactive antigen domain‐containing protein 1 (OCIAD1) interacts with prohibitin membrane scaffolds to protect TIMM17A from premature degradation, thereby promoting the TIMM17A‐containing variant of the TIM23 complex (Elancheliyan et al., 2024). Interestingly, OCIAD1 itself is a substrate of YME1L, creating a negative feedback circuit (MacVicar et al., 2019). A similar autoregulatory principle governs ROMO1, a TIM23 complex subunit, which is required for YME1L import into the IM but degraded by YME1L (Richter et al., 2019). These findings illustrate that YME1L coordinates its own assembly by modulating import via TIM23 complexes.

The turnover of subunits of the TIM23 complex has been identified as an important mechanism for coping with mitochondrial import stress. Preproteins that accumulate within translocase complexes and limit import can be selectively removed by the UPS as part of selective quality control pathways (den Brave et al., 2021; Pfanner et al., 2025; Song et al., 2021). Furthermore, the mitoproteases YME1L and OMA1 can clear import pores from stalled translocation intermediates (Coyne et al., 2023; Krakowczyk et al., 2024). However, clogging of import pores can cause remodeling of the mitochondrial translocase machinery to preserve mitochondrial homeostasis. YME1L functions as a critical sensor and effector of translocase occupancy. When protein import through TOM complexes stalls, YME1L degrades the TIMM17A and TIMM23 subunits of the TIM23 complex, thereby removing inactive translocases and mitigating the deleterious consequences of pore obstruction (Hsu et al., 2025). Many of these studies exploit exogenously expressed, artificial clogging proteins; however, pathogenic mutations in the mitochondrial carrier protein SLC25A4/ANT1 (A114P and A114P, A123D) cause the accumulation of the mutant proteins as translocation intermediates that interfere with protein import. This suggests that clogging of translocation pores is a disease mechanism (Coyne et al., 2023).

The loss of YME1L is associated with severe physiological deficiencies during embryonic development and in different tissues of mice (Hartmann et al., 2016; Sprenger et al., 2019; Wai et al., 2015; Wani et al., 2022; Zhou et al., 2023). Moreover, metabolic remodeling of mitochondria by YME1L‐mediated proteolysis maintains the neural stem and progenitor cell pool in adult mice (Wani et al., 2022) and facilitates the growth of pancreatic ductal adenocarcinoma (PDAC) tumors in xenograft models (MacVicar et al., 2019). Although YME1L is a multifaceted protease with quality control and regulatory functions (Ohba et al., 2020), it is an attractive hypothesis that the proteolytic remodeling of IM protein translocases, especially of the TIM23 complex, and the associated alterations in the mitochondrial proteome significantly contribute to these phenotypes.

## 5 TAILORING THE PROTEIN IMPORT MOTOR BY PROTEOLYSIS

The transport of preproteins through TIM23 complex into the matrix space or the IM is driven by the presequence‐translocase‐associated motor (PAM) complex at the inner surface of the IM. The HSP70 protein HSPA9 is a central component of this complex and interacts with the nucleotide exchange factors GRPEL1 and GRPEL2. These factors regulate protein import and folding in the matrix, but have nonredundant roles and differ significantly in their half‐lives (Figure 2). GRPEL1 is an essential, housekeeping nucleotide exchange factor that shows higher affinity for ADP‐bound HSPA9 than GRPEL2 and drives basal HSPA9 activity and protein import under normal conditions (Manjunath et al., 2024; Morizono et al., 2024). The short‐lived GRPEL2 is a cysteine‐rich, redox‐sensitive nucleotide exchange factor that fine‐tunes the chaperone system particularly under oxidative stress conditions (Konovalova et al., 2018; Tang et al., 2021; Yang et al., 2023). The mechanism by which GRPEL2 is degraded remains to be determined, but candidate proteases include the IM‐localized m‐AAA protease AFG3L2 or the matrix‐localized LONP1 protease.

**FIGURE 4:**
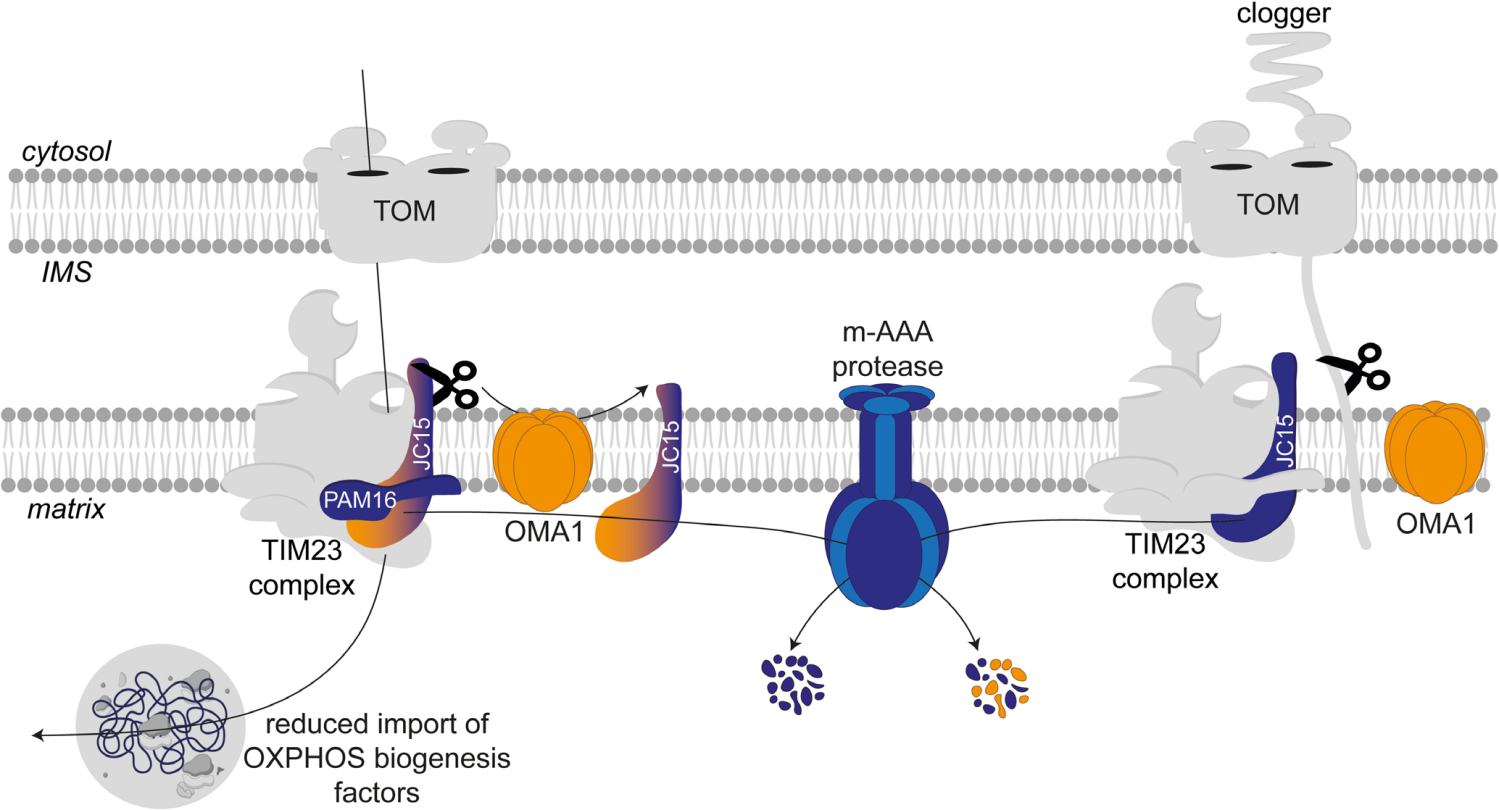
Proteolytic control of the PAM complex. Key components of the presequence translocase‐associated motor (PAM) complex, such as DNAJC15 and PAM16, are short‐lived and regulated by proteolysis. OMA1‐mediated processing facilitates degradation of DNAJC15 by the m‐AAA protease (PDB of AFG3L2: 6NYY), limiting the import of OXPHOS‐related proteins. Upon clogging of protein translocases by preproteins, OMA1 also cleaves stalled precursor proteins, allowing their release from mitochondria to the cytosol for proteasomal degradation. The concomitant reduction of DNAJC15, TIMM17A, and TIMM23 under these conditions suggests coordinated crosstalk between OMA1, the m‐AAA, and i‐AAA proteases in remodeling the translocase and clearing unoccupied TIM23 complexes.

Recently, direct evidence for proteolytic control of protein import under stress conditions was found for DNAJC15, another component of the PAM complex. The J‐domain containing proteins DNAJC15 and DNAJC19 (Pam18 in yeast) stimulate HSPA9, which is recruited to the TIM23 complex by TIMM44. DNAJC15 and DNAJC19 have redundant functions, but differ in their IMS‐exposed amino‐terminal region and have strikingly different apparent half‐lives, with DNAJC15 being rapidly degraded by the m‐AAA protease AFG3L2 (Kroczek et al., 2026) (Figure 4). The m‐AAA protease is a paralog of the i‐AAA protease in the IM but exposes its catalytic site to the matrix space rather than the IMS. Degradation of DNAJC15 by AFG3L2 is facilitated by the IM peptidase OMA1, which cleaves off 19 amino acids from the amino‐terminal region of DNAJC15 in the IMS (Kroczek et al., 2026). Numerous stress conditions, including OXPHOS deficiencies, were found to activate OMA1 (Baker et al., 2014; Bohovych et al., 2014; Bohovych et al., 2019; Kaser et al., 2003; Miallot et al., 2023; Murata et al., 2020; Ohba et al., 2020; Rainbolt et al., 2016; Zhang et al., 2014). This suggests that the accelerated degradation of DNAJC15 upon OMA1 cleavage reshapes mitochondrial protein import under stress conditions. The depletion of DNAJC15 is indeed associated with decreased protein levels of OXPHOS‐related proteins in mitochondria, decreased protein import rates and lowered respiratory activities (Kroczek et al., 2026). Thus, the OMA1‐AFG3L2‐dependent turnover of DNAJC15 fine‐tunes mitochondrial protein import and transiently limits OXPHOS biogenesis under stress, while preserving the basal import capacity. Non‐imported preproteins are diverted to the endoplasmic reticulum, where they trigger an unfolded protein response, and may serve as a protein reservoir until mitochondrial function recovers. Furthermore, it was shown that OMA1 modulates coenzyme Q biosynthesis by controlling the proteolytic processing of DNAJC15 to its short S‐form, which exerts an inhibitory effect on CoQ synthesis. Regulated OMA1‐dependent degradation of S‐DNAJC15 may therefore allow cells to tune CoQ levels in response to changing metabolic demands (Kroczek et al., 2026).

A proteomic survey in AFG3L2‐deficient cells identified DNAJC15 and PAM16 as substrates of the m‐AAA protease (Chandragiri et al., 2024). This suggests that the protease has broad effects on the remodeling of the mitochondrial import machinery (Figure 4). Since DNAJC15 was among the mitochondrial proteins most significantly decreased upon clogging of TOM complexes (Hsu et al., 2025), AFG32L2‐mediated degradation of DNAJC15 and PAM16 may also contribute to the proteolytic removal of surplus IM translocases during import stress. In yeast, the homologous LonP1 (Pim1) protease likewise targets Pam16 and Pam18, highlighting the evolutionary conservation of the proteolytic regulation of the mitochondrial import machinery (Boggula et al., 2025). Notably, AFG3L2 also determines the half‐live of OXA1L, a membrane insertase in the IM, suggesting even broader effects on mitochondrial protein biogenesis (Chandragiri et al., 2024).

## 6 RESHAPING THE MITOCHONDRIAL PROTEOME BY LIMITING IMPORT CAPACITY

The selective effect of DNAJC15 proteolysis on OXPHOS‐related proteins targeted to the matrix or IM raises the intriguing question of how the removal of a matrix‐localized PAM complex constituent affects the import of mitochondrial preproteins initiated at the OM. It is possible that an unrecognized specificity of protein translocases contributes to the selective effect of the loss of DNAJC15 on the mitochondrial proteome, but another scenario appears more plausible. The import of newly synthesized proteins and protein turnover determine the steady‐state levels of mitochondrial proteins. Interestingly, short‐lived proteins are enriched among the mitochondrial proteins affected by DNAJC15 proteolysis (Kroczek et al., 2026). This suggests that limiting the import capacity triggers the reshaping of the mitochondrial proteome allowing protein turnover by mitoproteases. Similarly, inhibiting cytosolic translation (Rainbolt et al., 2013) or clogging the mitochondrial import pore will result in decreased levels of short‐lived mitochondrial proteins (Hsu et al., 2025). Consistent with this hypothesis, DNAJC15 genetically interacts with short‐lived TIMM17A (but not TIMM17B) and depletion of TIMM17A (but not of TIMM17B) further decreases the levels of OXPHOS‐related proteins in mitochondria (Kroczek et al., 2026). TIMM17A is degraded by YME1L, which is activated during starvation or hypoxia. This indicates that different proteases modulate mitochondrial import capacity under various stress conditions.

## 7 AN EMERGING VIEW ON OMA1‐DRIVEN MITOCHONDRIAL STRESS RESPONSES

The stress‐activated peptidase OMA1 promotes the proteolysis of DNAJC15 and, consequently, the rewiring of the mitochondrial proteome. Previous studies have already identified OMA1 as a key regulator of mitochondrial stress responses. OMA1 cleaves OPA1, a dynamin‐like GTPase that mediates mitochondrial fusion and maintains cristae morphogenesis (Macvicar & Langer, 2016). Under stress, OMA1‐mediated OPA1 processing limits mitochondrial fusion, leading to fragmentation of the mitochondrial network via ongoing fission events and facilitating the removal of damaged mitochondria by mitophagy (Macvicar & Lane, 2014). OMA1 also cleaves DELE1 during protein import into functionally impaired mitochondria, thereby eliciting the ISR^mt^ (Fessler et al., 2020; Guo et al., 2020). This process broadly rewires the cellular and mitochondrial proteomes to cope with challenging metabolic demands. OMA1 has also recently been found to cleave AIFM1, an NADH oxidoreductase, upon prolonged mitochondrial stress (Nishigori et al., 2026). AIFM1 cleavage reduces its binding to OXPHOS subunits, thereby limiting OXPHOS activity and cell growth. This indicates complementary effects of DNAJC15 and AIFM1 cleavage.

**FIGURE 5:**
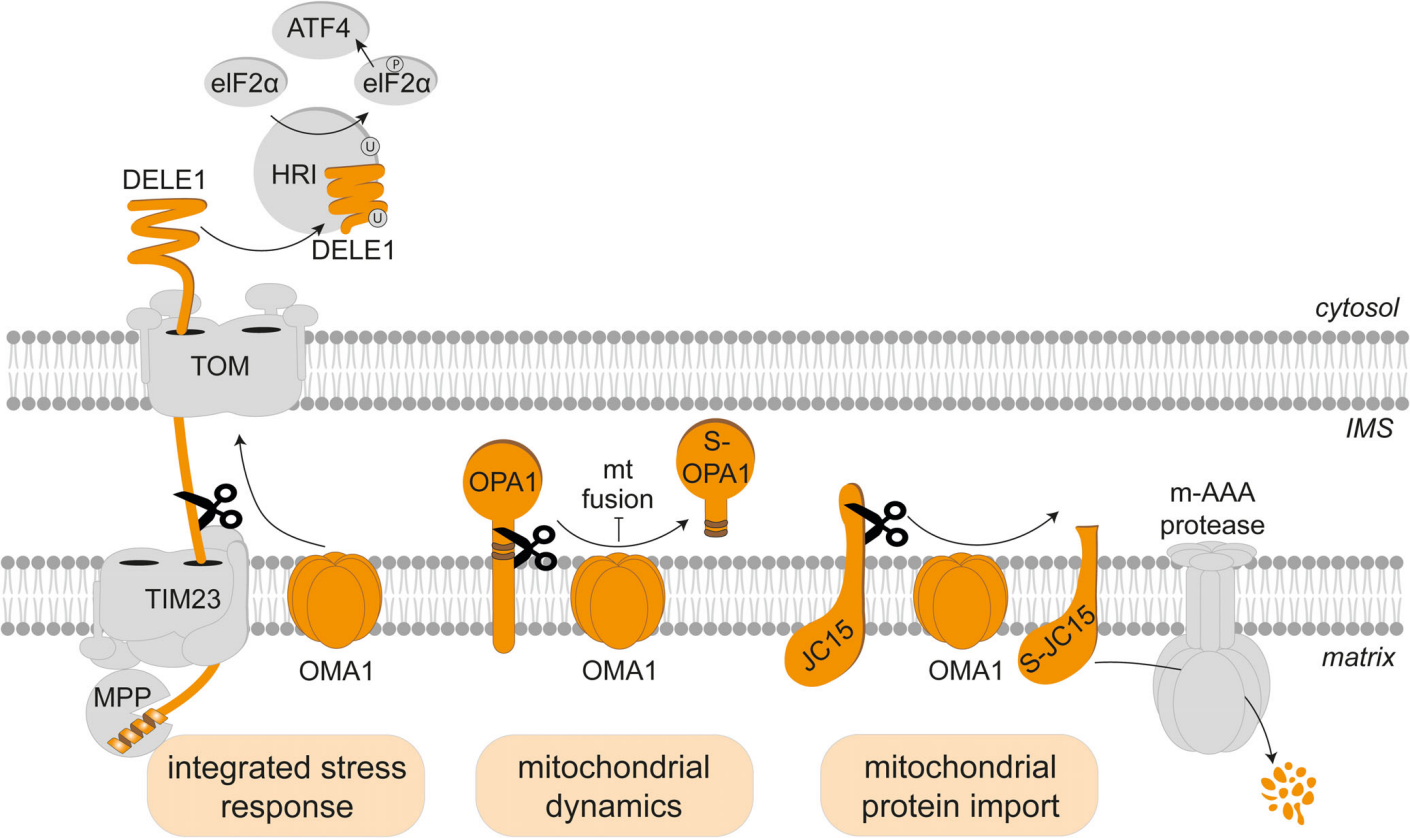
The OMA1‐mediated mitochondrial stress response. OMA1 orchestrates a multifaceted stress response that adapts the cellular metabolism and remodels mitochondrial architecture and function to maintain cellular homeostasis under adverse conditions, such as OXPHOS dysfunction. Under stress, OMA1 cleaves the dynamin‐like GTPase OPA1, limiting mitochondrial fusion and facilitating selective mitophagy. OMA1‐mediated DELE1 processing during import triggers the ISR^mt^, which rewires the cellular metabolism and transiently suppresses global translation to alleviate the proteostatic burden. Cleavage of DNAJC15 by OMA1 facilitates its degradation by AFG3L2, tailoring mitochondrial protein import and reducing the import of OXPHOS‐related proteins.

Thus, OMA1 orchestrates different regulatory circuits in response to mitochondrial stress ensuring cell survival (Figure 5). Available current literature suggests that different OMA1 functions occur independently of each other and, with the exception of AIFM1, have similar kinetics. However, increasing evidence indicates close cooperation with other mitoproteases. Proteolytic control of mitochondrial protein translocases is thus emerging as an important regulatory hub with broad effects on mitochondrial function. Future studies will identify the physiological conditions leading to the proteolytic rewiring of mitochondrial protein import and elucidate the determinants of protein import specificities. A better understanding of these regulatory circuits will allow to define the role of impaired protein import control in diseases associated with mitochondrial defects and mitoprotease deficiencies.

## AUTHOR CONTRIBUTIONS

**Lara Kroczek:** Conceptualization; visualization; writing – original draft; writing – review and editing. **Thomas Langer**: Conceptualization; writing – review and editing.

## FUNDING INFORMATION

This work was supported by the Deutsche Forschungsgemeinschaft (DFG) as part of the CRC1218 (grant number 269925409, project A01), and by institutional funds from the Max Planck Foundation. The funders had no role in the preparation of the manuscript.

## CONFLICT OF INTEREST STATEMENT

The authors declare that they have no competing financial or non‐financial interests related to the work.

## Data Availability

Data sharing not applicable to this article as no datasets were generated or analysed during the current study.
